# Enhancing fertilizer use efficiency in *Mentha spicata* L. through biochar-based integration with NPK in sandy soils

**DOI:** 10.1038/s41598-026-60697-2

**Published:** 2026-07-14

**Authors:** Yassin M. Soliman, Wagdi Saber Soliman

**Affiliations:** 1 Department of Horticulture, Faculty of Agriculture, Sohag University, Sohag, Egypt; 2 Horticulture Department, Faculty of Agriculture and Natural Resources, Aswan University, Aswan, 81528 Egypt

**Keywords:** *Mentha spicata* L., Biochar, NPK, Essential oil, Sustainable agriculture, Ecology, Ecology, Environmental sciences, Plant sciences

## Abstract

Sandy soils are widely distributed in Egypt and are characterized by poor physical properties and a limited capacity to retain irrigation water and nutrients, which severely constrains agricultural productivity. Addressing these limitations has become increasingly critical to ensure sustainable crop production under arid and semi-arid conditions. Therefore, this study aimed to evaluate the effectiveness of biochar as a soil amendment for improving growth performance, yield, nutrient accumulation, photosynthetic pigments, and essential oil production of *Mentha spicata* cultivated under sandy soil conditions over two successive seasons (2024–2025). The field experiment consisted of four treatments: untreated control, NPK at 5 g L^-1^, biochar at 12.5 ton ha^-1^, and combined NPK (5 g L^-1^) and biochar (12.5 ton ha^-1^). The results showed that biochar application, either alone or in combination with mineral fertilizer, significantly improved all vegetative growth parameters, fresh and dry herbage yields, essential oil percentage and yield, leaf macronutrient content (N, P, and K), and chlorophyll b concentration. Biochar applied alone consistently outperformed mineral fertilizer alone, while the combined biochar–NPK treatment produced the highest values for all measured traits, with improvements exceeding the additive effects of individual applications. These enhancements were closely associated with increased nutrient accumulation and improved nutrient use efficiency, reflecting the ability of biochar to retain nutrients, reduce leaching losses, and synchronize nutrient availability with plant demand. The synergistic interaction between biochar and mineral fertilization highlights the potential of biochar as a sustainable soil amendment for improving productivity, quality, and resource-use efficiency of mint grown in low-fertility sandy soils.

## Introduction

Sandy soils are extensively distributed worldwide and are increasingly exploited to meet the rising demand for food, feed, fiber, and bio-based resources driven by population growth and urban expansion^1^. These soils are characterized by a sand content exceeding 70%, which confers distinct physical and chemical limitations, including rapid water infiltration, low water- and nutrient-holding capacity, high susceptibility to drought stress, and vulnerability to wind erosion. Consequently, sandy soils are inherently poor in fertility and pose serious constraints to sustainable agricultural production under conventional management practices^2^. Addressing these limitations has therefore become a critical priority to ensure efficient and resilient crop production in arid and semi-arid regions.

Mineral fertilizers remain indispensable for modern agriculture due to their pivotal role in supporting plant growth and productivity. Nitrogen (N) is a fundamental constituent of amino acids, proteins, and enzymes, and its application has been shown to markedly increase biomass accumulation and essential oil yield in several medicinal and aromatic plants^3,4^. Phosphorus (P) is crucial for energy transfer, photosynthesis, and nucleic acid synthesis, while potassium (K) regulates enzymatic activity, nitrogen metabolism, and the synthesis of essential metabolites, collectively contributing to enhanced yield and oil content^5^. However, the excessive and inefficient use of chemical fertilizers has resulted in serious environmental challenges, including soil degradation, nutrient leaching, groundwater contamination, and greenhouse gas emissions. Thus, while mineral fertilizers are indispensable for sustaining crop production, their environmental and economic drawbacks necessitate the development of complementary strategies that improve fertilizer use efficiency and reduce ecological risks^6^.

The degradation of sandy soils is further exacerbated by unsustainable agricultural practices, which have been identified as major drivers of soil biodiversity loss, declining fertility, and reduced ecosystem functioning^7,8^. In this context, the adoption of sustainable agricultural strategies is essential not only to preserve soil quality but also to secure stable crop yields and ensure the production of safe and healthy food^9^. Sustainable soil management integrates biological, physical, and chemical processes to enhance soil functioning by stimulating beneficial microbial activity, promoting organic matter decomposition, suppressing soil-borne pathogens, inactivating toxic compounds, and improving the recycling of nutrients, water, and energy within agroecosystems^10–12^.

Among the proposed solutions, biochar has emerged as a promising, environmentally friendly, and cost-effective soil amendment. Biochar is a carbon-rich material produced through the thermal decomposition of biomass under limited or oxygen-free conditions. Owing to its high carbon content (65–90%), large surface area, porous structure, and strong adsorption capacity, biochar exhibits unique physicochemical properties that are particularly beneficial for degraded soils^13,14^. Numerous studies have demonstrated that biochar application can improve soil structure, enhance microbial activity, increase nutrient retention and availability, and ultimately promote plant growth and productivity^14,15^. Furthermore, biochar contributes to the stabilization and accumulation of soil organic carbon, a key determinant of soil fertility that has been steadily declining due to intensive agriculture and climate change^16,17^.

In sandy soils, biochar has been shown to enhance the availability and uptake of essential nutrients, particularly N, P, and K, while improving soil moisture retention, photosynthetic efficiency, and biomass production^18–21^. Nevertheless, the effectiveness of biochar is strongly influenced by its feedstock, production conditions, and application rate. While moderate application rates generally stimulate plant growth and soil quality, excessive biochar additions may adversely affect soil physicochemical properties, microbial biomass, and crop performance^22–24^. Optimal biochar application rates are commonly reported to range between 1 and 4%, whereas higher rates (> 5%) may hinder seed germination or restrict nutrient availability^25,26^. In addition, biochar has been reported to suppress several soil-borne diseases by enhancing soil microbial diversity, stimulating plant defense responses, and reducing pathogen populations^27–30^.

The integration of organic soil amendments, such as biochar, has therefore been recognized as an effective approach to improving soil moisture retention, microbial activity, and nutrient cycling in sandy soils^31,32^. Recent studies have also highlighted the positive effects of biochar on the growth and productivity of medicinal and aromatic plants, including *Mentha longifolia*^33^, as well as forage crops cultivated under marginal soil conditions^34^.

Spearmint (*Mentha spicata* L.), a perennial aromatic herb belonging to the Lamiaceae family, is widely cultivated worldwide, including in Egypt, due to its high economic and medicinal value^35^. Its fresh and dried leaves are commonly used as herbal teas, spices, and flavoring agents, while its herbage, extracts, and essential oil (EO) have long been employed in traditional medicine. Spearmint EO is extensively utilized in the food, pharmaceutical, and cosmetic industries, particularly in chewing gum, toothpaste, mouthwash, confectionery, fragrances, and medicinal formulations, owing to its distinctive aroma and bioactive properties^36,37^. Chemically, spearmint EO is rich in biologically active compounds, including phenolic antioxidants, cholinesterase inhibitors, and antifungal and antiproliferative agents^38^.

Despite the growing body of research on the individual effects of biochar or mineral fertilizers, limited information is available on the combined application of biochar and NPK fertilizers for spearmint cultivation under sandy soil conditions. We hypothesize that biochar enhances mineral fertilizer use efficiency in sandy soils as well as that combined biochar–NPK application results in superior plant performance compared with single applications. Therefore, the present study aimed to evaluate the individual and synergistic effects of these inputs on plant growth, productivity, essential oil yield, and chemical composition of *Mentha spicata* grown in sandy soils, with a focus on improving fertilizer use efficiency and promoting environmentally sustainable production systems.

## Materials and methods

### Experimental site and growth conditions

A field experiment was conducted during two consecutive growing seasons (2024 and 2025) at the Agricultural Research Station, Al-Marashda, Qena Governorate, Egypt. Prior to the establishment of the experiment, composite soil samples were collected from the experimental field and analyzed for their physical and chemical properties following the standard procedures^39,40^. The initial soil characteristics are presented in Table 1. The soil texture was sandy soil with 1.35% organic matter (OM). The recorded OM value may be attributed to previous organic inputs and residue accumulation under irrigated field conditions, which can enhance carbon stabilization even in coarse-textured soils.

Uniform seedlings of spearmint (*Mentha spicata* L.), approximately 7 cm in height, were obtained from a certified private medicinal plant nursery in Qena Governorate, Egypt. Seedlings were transplanted on 15th February in both growing seasons. The experiment was arranged in a randomized complete block design with three replicates. Four treatments were evaluated: (i) untreated control, (ii) foliar application of NPK fertilizer (5 g L^-1^), (iii) soil application of biochar (12.5 ton ha^-1^), and (iv) combined application of NPK (5 g L^-1^) and biochar (12.5 ton ha^-1^).

### Biochar and fertilizer application

The biochar used in this study was a porous, sponge-like carbonaceous material produced by pyrolysis of olive and rice residues at 350–400 °C. The biochar was supplied by Power Tech Company (Cairo, Egypt), and its chemical properties are presented in Table 2. Biochar was uniformly incorporated into the soil during land preparation prior to transplanting.

A balanced water-soluble NPK fertilizer (20–20–20) was applied as a foliar spray. Foliar applications were carried out three times per cutting cycle. The first spray was applied 21 days after transplanting or harvesting, followed by two additional applications at 10-day intervals.

Each experimental sub-plot measured 1.8 × 1.8 m and consisted of three rows containing 18 plants. Plant spacing was maintained at 60 cm between rows and 30 cm within rows. All recommended agronomic practices for spearmint cultivation were uniformly applied throughout the growing period.

### Harvesting schedule

Spearmint plants were harvested three times per season at 45-day intervals. The first harvest was conducted in mid-April, the second in late May, and the third in mid-July during both experimental seasons.

**Table 1:** Physical and chemical properties of the soil under study.

Character	Value	Character	Value
Texture analysis		Soluble cations (meq/100 g soil)	
Clay %	8.5	Ca^++^	1.84
Silt %	6.5	Mg^++^	0.86
Sand %	85.0	Na^+^	2.92
Texture grade	sandy	K^+^	1.43
Total CaCO_3_ (%)	1.51	Soluble anions (meq/100 g soil)	
E.C. dS/m (1:5) soil extract	3.13	Cl^-^	3.19
pH (1:2.5 soil suspension)	8.2	HCO_3_^-^	2.45
Organic matter (%)	1.35	SO_4_^-^	1.41
		Total nitrogen (%)	0.33
		Available phosphorus (mg g^-1^)	0.091
		Exchangeable potassium mg g^-1^	0.048

**Table 2:** Chemical analysis of the used biochar in the study.

Component	pH	EC	Total *N*	Organic carbon	Ash	K_2_O	*P*_2_O_5_	SiO_2_
Value (%)	7.8	2.5 dS m^-1^	0.8%	42.5%	1.7%	26.0%	3.6%	25.0%

### Measurements and analytical methods

#### Growth and yield components

Vegetative growth data were recorded at each harvest and included plant height (cm), number of branches per plant, number of leaves per plant, and leaf area (cm²).

Herb yield was determined as fresh and dry weights. Plant samples were directly weighed after harvest to determine the fresh weight, then oven-dried at 70 °C until a constant weight was achieved to determine dry matter. Herb yield per hectare was calculated by multiplying the mean fresh or dry weight per plant by plant density per hectare (based on spacing of 60 × 30 cm), and then converting to kg ha^-1^.

#### Essential oil extraction and yield

Essential oil was extracted from 100 g of fresh herbage using hydro-distillation for 2 h. The essential oil content was expressed as milliliters of oil per 100 g fresh weight (%), and essential oil yield per plant was calculated following the method described by Guenther^41^.

#### Photosynthetic pigments

Photosynthetic pigments, including chlorophyll a, chlorophyll b, and carotenoid, were extracted from fresh leaf samples using dimethyl formamide [HCON(CH₃)₂] and stored overnight at 5 °C. Absorbance was measured using a spectrophotometer (Shimadzu UV-12002) at wavelengths of 663, 647, and 470 nm. Pigment concentrations were calculated according to Nornai^42^ using the following equations:

Chlorophyll a = 12.70 A₆₆₃ − 2.79 A₆₄₇

Chlorophyll b = 20.76 A₆₄₇ − 4.62 A₆₆₃

Total carotenoids = [1000 A₄₇₀ − (3.72 Chl a + 104 Chl b)]/229

#### Nitrogen, phosphorus, and potassium content

Leaf nitrogen (N), phosphorus (P), and potassium (K) percentages were determined following the standard analytical procedures recommended by ICARDA^43^. Leaf samples were wet-digested with concentrated H_2_SO_4_:H_2_O_2_. Nitrogen content was measured using the semi-micro Kjeldahl method, phosphorus content was measured using the vanadate-molybdate-yellow method, and the potassium content was measured using a flame photometer.

### Statistical analysis

Data obtained from both growing seasons were subjected to statistical analysis using *Statistix* software (version 8.1). The obtained data were statistically analyzed using the F-test^44^. Differences among the interaction treatments were assessed using Tukey’s honestly significant difference (HSD) test, following the procedure described by Gomez and Gomez^45^.

## Results

### Plant growth characteristics

**Fig. 1:**
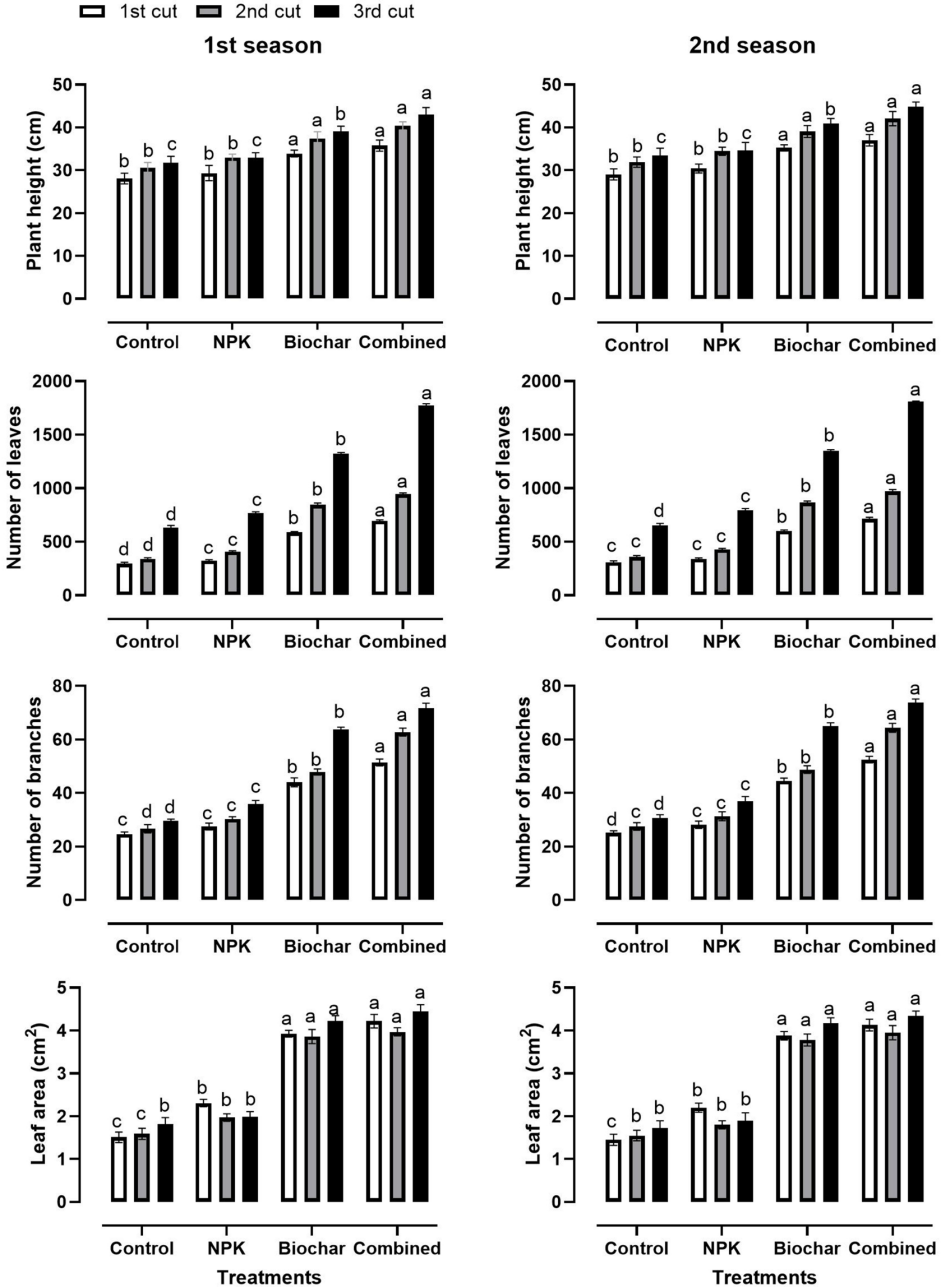
Plant growth characteristics of spearmint as affected by mineral NPK and biochar alone or in combination during the 2024 and 2025 seasons. Different letters represent significant differences among treatments.

Analysis of variance (ANOVA) revealed significant effects of mineral fertilizer, biochar, and their combination on vegetative growth parameters, including plant height, number of leaves per plant, number of branches per plant, and leaf area, across all harvests and during both growing seasons (Fig. 1). The combined application of biochar and mineral fertilizer consistently produced the highest values for all growth traits, followed by biochar alone, whereas the control treatment recorded the lowest values.

Plant height was significantly increased under the combined biochar and mineral fertilizer treatment, reaching 35.8–43.0 cm compared with 28.1–31.8 cm in the control during the first season, and 37.0–41.0 cm compared with 29.0–32.1 cm in the second season. Similarly, the number of leaves per plant ranged from 695 to 1773 leaves in the first season and from 715 to 1348 leaves in the second season under the combined treatment, compared with 295–632 and 309–504 leaves under the control in the first and second seasons, respectively. The number of branches per plant also increased markedly, ranging from 5 to 72 branches in the first season and 53–65 branches in the second season under the combined treatment, compared with 25–30 branches in the control. Leaf area followed a similar trend, ranging from 4.22 to 4.44 cm^2^ in the first season and 4.13–4.18 cm^2^ in the second season under the combined treatment, whereas the control recorded values of 1.51–1.82 cm^2^ and 1.45–1.64 cm^2^ in the first and second seasons, respectively.

### Herb yield characteristics

**Fig. 2:**
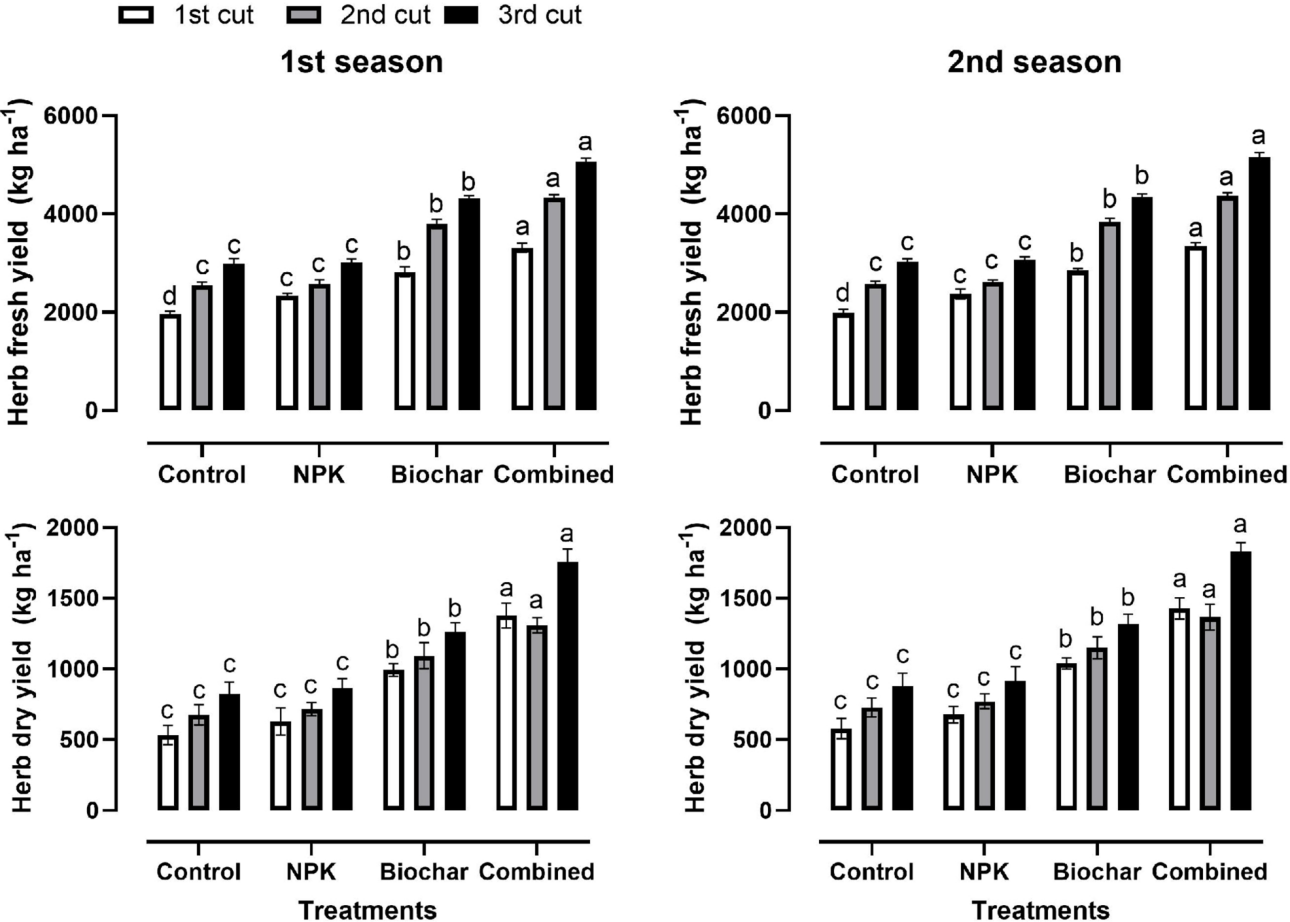
Herb yield characteristics of spearmint as affected by mineral NPK and biochar alone or in combination during the 2024 and 2025 seasons. Different letters represent significant differences among treatments.

In the same manner of vegetative growth, the application of mineral fertilizer, biochar, or their combination resulted in significant increases in both fresh and dry herb yield (Fig. 2). The highest yields were obtained from the combined biochar and mineral fertilizer treatment, followed by biochar alone, while the lowest yields were observed in the control. Fresh herb yield under the combined treatment reached 3311–5050 kg ha⁻¹ compared with 1972–2994 kg ha⁻¹ in the control during the first season, and 3350–4340 kg ha⁻¹ compared with 1990–2940 kg ha⁻¹ during the second season. Dry herb yield under the combined treatment ranged from 1377 to 1757 kg ha⁻¹ in the first season and 1428–1711 kg ha⁻¹ in the second season, whereas the control produced only 532–827 and 578–758 kg ha⁻¹ in the first and second seasons, respectively.

### Essential oil percentage and yield

**Fig. 3:**
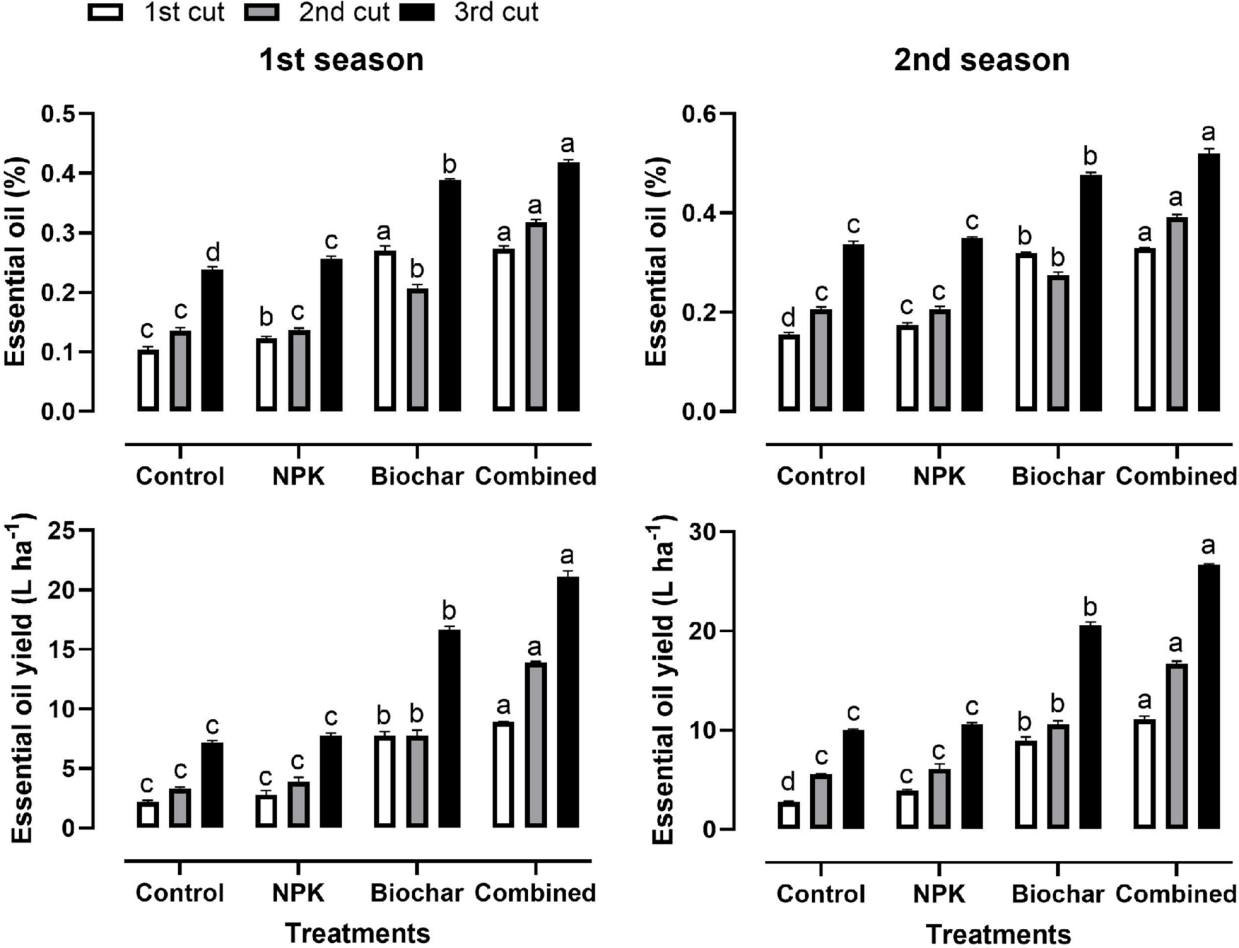
Essential oil percentage and yield of spearmint as affected by mineral NPK and biochar alone or in combination during the 2024 and 2025 seasons. Different letters represent significant differences among treatments.

Conversely, essential oil percentage and essential oil yield were significantly and positively affected by biochar application. The highest oil percentage and oil yield were obtained under the combined biochar and mineral fertilizer treatment, followed by biochar alone, while the control treatment recorded the lowest values (Fig. 3). Oil percentage under the combined treatment ranged from 0.27 to 0.42% compared with 0.10–0.24% in the control during the first season, and from 0.33 to 0.48% compared with 0.16–0.46% in the second season. Essential oil yield reached 9–21 and 11–21 L ha^− 1^ under the combined treatment in the first and second seasons, respectively, compared with 2–7 and 3–8 L ha⁻¹ for the control.

### Photosynthetic pigments and macronutrients content

**Fig. 4:**
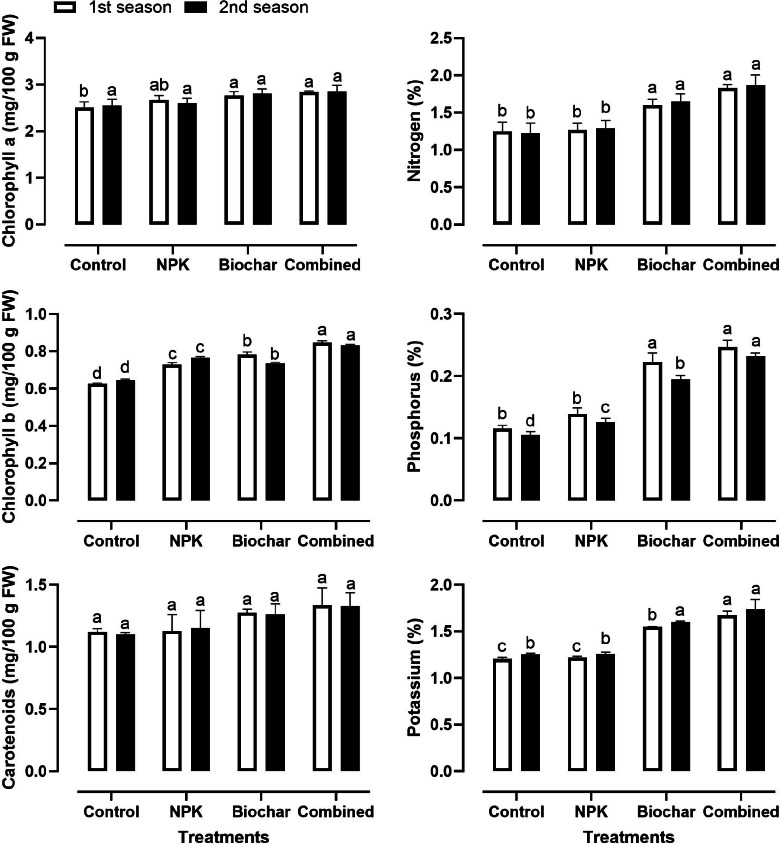
Photosynthetic pigments and macronutrients content of spearmint as affected by mineral NPK and biochar alone or in combination during the 2024 and 2025 seasons. Different letters represent significant differences among treatments.

Regarding photosynthetic pigments, ANOVA indicated that mineral fertilizer, biochar, and their combination had a significant effect on leaf chlorophyll *b* content, whereas their effects on chlorophyll *a* and carotenoid contents were not statistically significant (Fig. 4). The highest chlorophyll *b* values were recorded under the combined mineral fertilizer and biochar treatment (0.83–0.85 mg 100 g⁻¹) compared with the control (0.63–0.65 mg 100 g⁻¹).

In contrast, leaf macronutrient contents were significantly influenced by mineral fertilizer, biochar, and their combined application. The combined treatment resulted in the highest concentrations of nitrogen (N), phosphorus (P), and potassium (K), followed by biochar alone, while the control exhibited the lowest values (Fig. 4). Under the combined treatment, N, P, and K contents reached 1.83%, 0.25%, and 1.67%, respectively, in the first season, and 1.87%, 0.32%, and 1.60% in the second season. In comparison, the control treatment recorded 1.25%, 0.12%, and 1.21% in the first season and 1.23%, 0.11%, and 1.80% in the second season for N, P, and K, respectively.

### Correlation coefficients

**Fig. 5:**
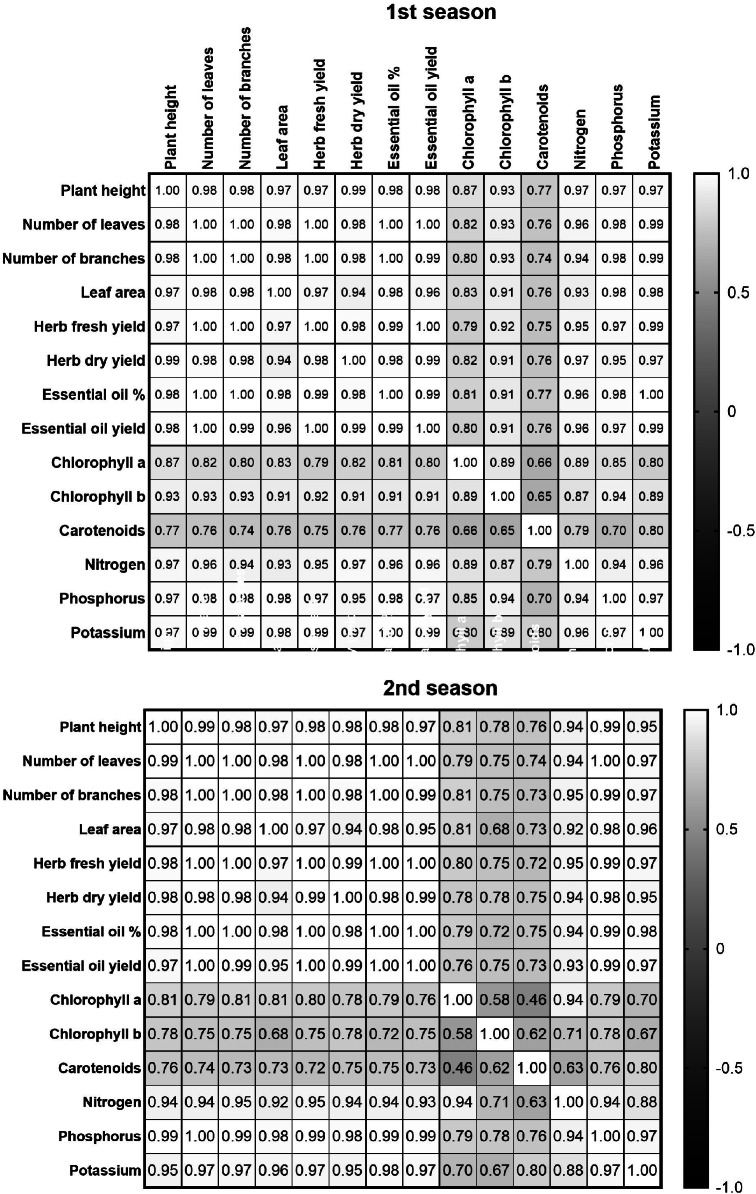
Correlation coefficients of growth traits, yield components, essential oil percentage and yield, photosynthetic pigments and nutrients content.

Correlation analysis revealed the presence of strong, positive, and highly significant relationships among all studied traits. These parameters showed consistent positive increases in response to biochar application and, more markedly, to the combined application of biochar and mineral fertilizer. In most cases, correlation coefficient (r) values exceeded 90% among growth attributes, yield components, quality traits, and macronutrient contents (Fig. 5), indicating a high degree of interdependence among these variables.

In contrast, weaker correlations were observed for chlorophyll *a* and carotenoid contents, which exhibited only slight and statistically non-significant variations among the different treatments. This suggests that, unlike other growth, yield, and nutrient parameters, chlorophyll *a* and carotenoids were less responsive to biochar and mineral fertilizer applications and contributed less to the overall variation among treatments.

## Discussion

The present study demonstrated consistent and progressive improvements in growth, yield, nutrient accumulation, photosynthetic performance, and essential oil production of *Mentha spicata* in response to mineral fertilizer, biochar, and their combined application. Notably, biochar applied alone produced substantially greater improvements than mineral fertilizer alone, while the combined application consistently exceeded the additive effects of the individual treatments. This clear synergistic response highlights the pivotal role of biochar in enhancing nutrient use efficiency and sustaining crop productivity under sandy soil conditions.

Among yield components, fresh and dry herbage yields—the principal economic traits of mint—responded strongly to biochar application. Fresh herbage yield increased by only about 6% under mineral fertilizer alone compared with the control, whereas biochar alone resulted in an increase of approximately 45%. The combined application further amplified this response to nearly 69%, surpassing the cumulative effects of each input applied separately. A similar trend was observed for dry herbage yield, which increased by 8 and 9% with mineral fertilizer alone, by 61 and 65% with biochar alone, and by approximately 112 and 118% under the combined treatment in the first and second season, respectively. These pronounced yield gains demonstrate that biochar substantially improves the efficiency with which mineral nutrients are converted into biomass, making it more effective than mineral fertilization alone.

The superior performance of biochar-amended treatments closely corresponded with marked improvements in leaf macronutrient accumulation. Compared with the control, mineral fertilizer alone increased leaf N, P, and K contents by approximately 4%, 20%, and 1%, respectively. In contrast, biochar application alone enhanced N, P, and K accumulation by 31%, 97%, and 28%, respectively, while the combined application further increased these values to 50%, 117%, and 39%. These findings unequivocally confirm that biochar enhances nutrient uptake and retention, either directly or through improving fertilizer use efficiency when combined with mineral inputs, thereby reducing nutrient losses typically associated with sandy soils.

The underlying mechanisms driving this enhanced nutrient use efficiency are strongly linked to the unique physicochemical properties of biochar. Owing to its high porosity, large specific surface area, and elevated cation exchange capacity, biochar functions as a nutrient reservoir, retaining applied N, P, and K in plant-available forms and synchronizing their release with crop demand^46,47^. This buffering capacity minimizes leaching losses and ensures sustained nutrient availability, which explains the significantly higher leaf nutrient concentrations and biomass yields recorded under biochar treatments.

Beyond nutrient retention, biochar application improved soil physical and biological properties, further enhancing nutrient acquisition. Increased soil organic carbon inputs stimulated microbial activity in the rhizosphere, accelerating nutrient mineralization and improving nutrient cycling efficiency^48,49^. Biochar-derived carbon has been shown to promote microbial biomass and enzymatic activity, facilitating phosphorus solubilization and improving nitrogen availability^46,50^. These improvements in soil biological functioning likely contributed to the enhanced nutrient uptake efficiency and sustained plant growth observed in the present study.

Biochar is known to improve soil water-holding capacity^51^, which may have contributed to the further reinforced nutrient use efficiency by reducing nutrient leaching and maintaining favorable moisture conditions for root activity and nutrient transport. This effect is particularly critical in sandy soils, which are inherently characterized by poor aggregation, low organic matter content, and limited nutrient- and water-holding capacity^52,53^. The improved water–nutrient balance under biochar application likely supported sustained nutrient absorption, resulting in significant increases in plant height, leaf number, branching, and leaf area, as previously reported for aromatic crops^54,55^.

Improvements in nutrient status were also reflected in enhanced photosynthetic performance, particularly the significant increase in chlorophyll *b* content under biochar-amended treatments. Adequate and sustained nitrogen availability is essential for chlorophyll synthesis and photosynthetic enzyme activity, while improved potassium availability contributes to chloroplast stability and enzymatic activation, thereby reducing pigment degradation^37,56,57^. These combined effects explain the strong association between enhanced nutrient accumulation, improved photosynthetic pigment concentration, and increased biomass production.

The benefits of improved nutrient use efficiency extended beyond biomass production to quality-related traits, particularly essential oil percentage and yield. Essential oil percentage increased by only 4–8% under mineral fertilizer alone, but by 54–81% under biochar alone and by 78–111% under the combined application. Similarly, essential oil yield increased by 12–13% with mineral fertilizer alone, by 118–152% with biochar alone, and by 197–244% under the combined treatment. Biochar enhances essential oil production by improving nutrient availability (especially N and K), increasing photosynthetic efficiency, and promoting carbon allocation toward secondary metabolism. Additionally, improved soil microbial activity and enzyme stimulation may enhance terpene biosynthesis pathways^51^. These substantial increases suggest that biochar-enhanced nutrient availability and photosynthetic efficiency improved carbon assimilation and partitioning toward secondary metabolite biosynthesis. Improved potassium nutrition associated with biochar treatments may have further stimulated enzymatic pathways involved in terpene synthesis, thereby increasing essential oil accumulation^58–61^.

The synergistic interaction between biochar and mineral NPK fertilization represents one of the most important outcomes of this study. While mineral fertilizers supplied readily available nutrients, biochar enhanced their retention, availability, and uptake efficiency, creating a more balanced and resilient nutrient supply system. This synergy resulted in improved rhizosphere conditions, enhanced physiological performance, and superior yield and quality attributes compared with either input applied alone.

These findings emphasize that biochar acts primarily as a nutrient efficiency enhancer rather than merely a nutrient source. Its integration with mineral fertilization represents a sustainable agronomic strategy capable of improving productivity, reducing nutrient losses, and enhancing soil health in sandy soils. Such an approach aligns with current efforts to promote sustainable agriculture by maximizing resource-use efficiency while minimizing environmental impacts, particularly in fragile agroecosystems prone to nutrient depletion and leaching.

## Conclusions

Biochar significantly improved vegetative growth, biomass yield, nutrient uptake, and essential oil production of *Mentha spicata* under sandy soil conditions. The combined application of biochar and NPK fertilizer produced the highest performance, indicating a strong synergistic effect. These results confirm that biochar enhances fertilizer efficiency by improving nutrient retention and availability in sandy soils. Integrating biochar with mineral fertilization therefore represents an effective and sustainable strategy for improving productivity and quality of aromatic crops. However, optimization of biochar application rates and evaluation of long-term impacts on soil properties and crop performance require further investigation.

## Data Availability

All relevant data are included in the manuscript, and any other information can be given by the corresponding author.
